# Developing a medium combination to attain similar glycosylation profile to originator by DoE and cluster analysis method

**DOI:** 10.1038/s41598-021-86447-0

**Published:** 2021-03-29

**Authors:** Jian Xu, Zhihui Shao, Zhanqing Wang, Yingfeng Huang, Xun Zou, Yaling Shen

**Affiliations:** 1 State Key Laboratory of Bioreactor Engineering, Shanghai Collaborative Innovation Center for Biomanufacturing Technology, East China University of Science and Technology, Shanghai, 200237 China; 2 CAS Key Laboratory of Synthetic Biology, CAS Center for Excellence in Molecular Plant Sciences, Institute of Plant Physiology and Ecology, Chinese Academy of Sciences, Shanghai, China; 3 Process Development Department, Dragon Sail Pharmaceutical, Shanghai, China; 4 R&D Laboratories, Dragonboat Biopharmaceutical, Shanghai, China; 5 Shanghai Sanjin Bioscience and Technology, Shanghai, China

**Keywords:** Biological techniques, Biotechnology

## Abstract

Glycosylation is critical for monoclonal antibody production because of its impact on pharmacokinetics and pharmacodynamics. Modulation of glycan profile is frequently needed in biosimilar development. However, glycosylation profile is not a single value like that of cell culture titer, hence making it challenging for the Design of Experiment (DoE) methodology to be directly applied. In this study, a Her2-binding antibody was developed as a biosimilar to Herceptin. Cluster analysis was introduced to demonstrate the similarity of glycan profiles between the samples and the reference with specific value—distance. The glycosylation was subsequently optimized with the DoE method. Basal medium and feed medium were found to be the significant factors to the glycosylation pattern. Moreover, a combination of medium and feed strategy was developed to attain the most similar glycoprotein molecule to that of the originator biologic drug. This study may provide an additional option to evaluate multivariable factors and assess biosimilarity and/or comparability in monoclonal antibody production.

## Introduction

Glycosylation is one of the most vital critical quality attributes (CQA) during therapeutics antibody development due to the fact that its impact on the pharmacokinetics (PK) and pharmacodynamics (PD) can be significant in most cases. Guidelines for biosimilar development from several regulatory agencies across the world including FDA and EMA all consider glycoform similarity as one of the most important requirements^1–6^. It follows that the glycosylation attribute would normally be under stringent control during Chemistry Manufacturing and Controls (CMC) development and manufacture phases.

There are two types of protein glycosylation: serine/threonine-linked glycosylation (O-linked) and asparagine-linked glycosylation (N-linked). For antibodies, O-linked glycosylation is seldom seen^7,8^. Only some Fc-fusion partner molecules such as etanercept and B cell activating factor receptor 3 (BR3)-Fc possess O-linked glycans^9,10^. The N-linked glycan is attached at the Asn^297^ position in the heavy chain of therapeutic antibodies or derivatives where an asparagine (Asn)-X-Ser/Thr (X denotes any amino acid except Pro) consensus sequence^11^ is present.

**Figure 1:**
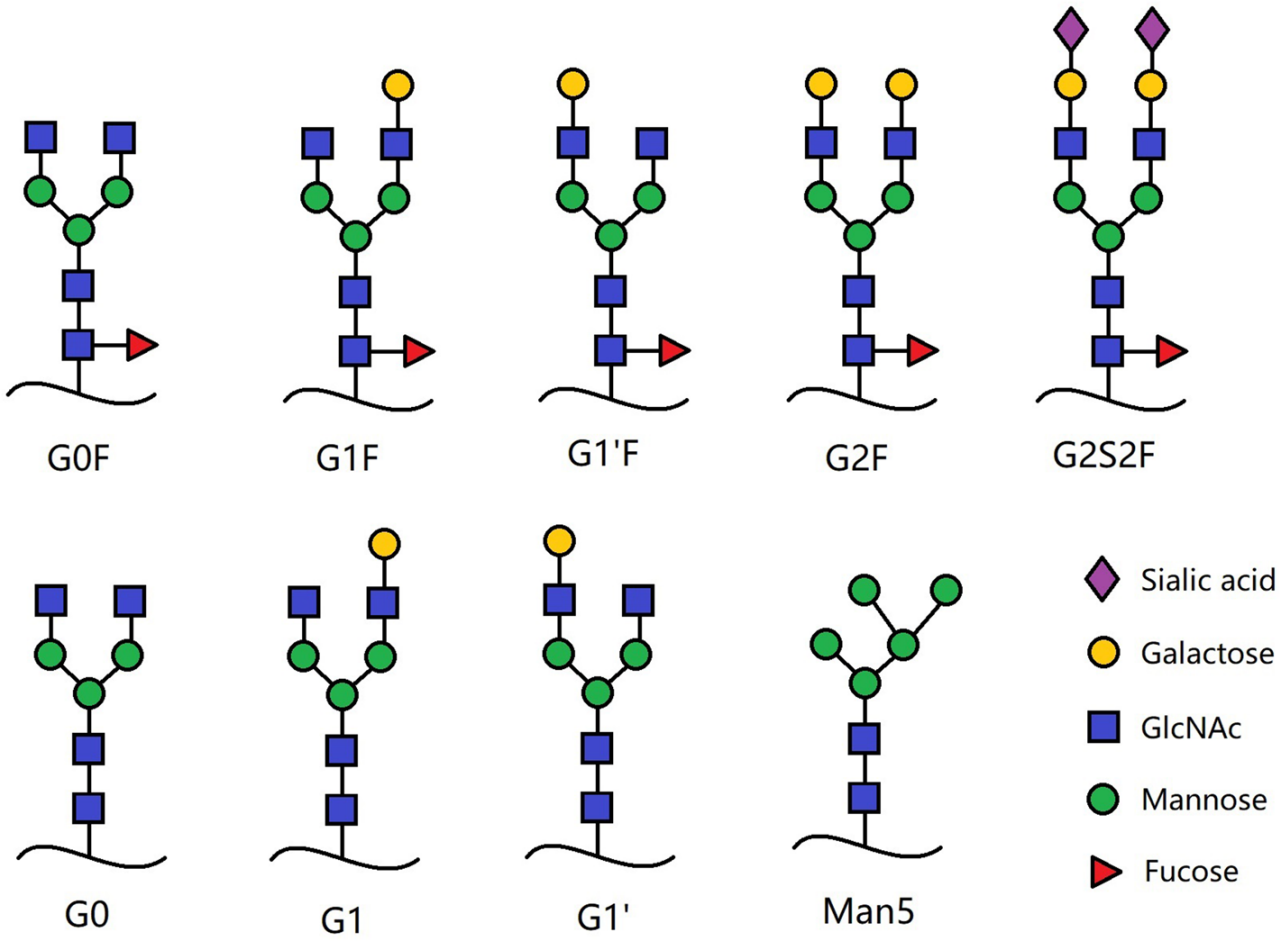
Major N-linked glycoforms of mAb therapeutics.

The N-linked glycans of human IgGs are typically biantennary complex structures. Two N-acetylglycosamine (GlcNAc), three mannose, and two GlcNAc residues compose the conserved core structure and two GlcNAc residues are β-1,2 linked to α-6 mannose and α-3 mannose, forming two antennae. Additional fucose (Fuc), galactose (Gal), bisecting GlcNAc, and sialic acid including N-acetylneuraminic acid (NANA) and N-glycolylneuraminic acid (NGNA) residues are added to the core, depending on the host glycosylation machinery. The major N-linked glycoforms of mAb therapeutics are shown in Fig. 1. The glycan structure showe considerable heterogeneity with more than several hundred glycoforms because of the random pairing of heavy-chain glycans with different structures^11,12^. Beside the expression hosts such as Chinese hamster ovary (CHO) cells and murine myeloma cells NS0 and SP2/0, cell culture conditions, medium and additives also affect antibody glycosylation as reported^13^ previously.

Glycosylation of therapeutic antibodies substantially affect PK profile, efficacy, and in some cases, antigenicity. Glycosylated antibodies with terminal high-mannose glycans exhibited fast clearance from the bloodstream^14–17^. Wright and coworkers revealed that IgG with glycans terminated with Man5 cleared significantly faster than those with more complex glycosylation such as G0F, G1F and G2F^18,19^. For Fc-fusion proteins, glycosylation played a more important role in determining the in-vivo clearance. Liu et al. demonstrated that the exposure of a humanized yeast-produced TNFαRII-Fc-fusion molecules were positively correlated to the quantity of the sialylation on the receptor molecule with higher sialic acid content resulting in higher exposure^20^. The glycosylation of humanized or fully human antibodies has critical impact on the Fc receptor-mediated effector functions. Many studies demonstrated defucosylated marketed mAbs such as rituximab and trastuzumab increased antibody-dependent cell-mediated cytotoxicity (ADCC) activities by at least two orders of magnitude in humans in vitro^21–23^. The first patent related to afucosylated IgG enhancing ADCC was published in 2000^24^. An afucosylated IgG exhibited a 50-fold increase in binding to the Fcγ RIIIa receptor, and ADCC activity was greatly enhanced too^22^. The terminal Gal played an important role in complement-dependent cytotoxicity (CDC) activity. For rituximab, CDC activity and the affinity to C1q increased by two folds when Gal content increased in the heavy chain^25^. Furthermore, glycan composition could be the factor to cause immunogenicity. High-mannose-type N-glycans is highly immunogenic in human^26^. Antibodies produced in mouse myeloma cells may contain α-Gal epitope (Galα1-3Galβ1-4GlcNAc-R), which could be immunogenic in human. Considering these significant impacts of glycosylation, the glycan profile should be well-studied and controlled during the manufacturing process and the development of a biosimilar product.

Since the effects of cell line, culture mode, medium and manufacturing conditions on the antibody glycan profiles were reported extensively, the effects of basal/feed medium combination and galactose addition will be on the focus in this study. Mammalian cell culture medium are made up of either chemically defined or serum-free mixtures of at least 50–100 different components. The concentration of glucose, amino acid, vitamin, metal ion and lipids supplements can play a role in glycosylation control. 3 kinds of basal medium and 2 kinds of feed were tested in this study. Chee Furng et al*.* found that the glucose concentration below 0.7 mM led to decrease in sialylation levels and increase in both hybrid type and high mannose type glycans in CHO fed-batch cultures producing IFN-γ^27^. Amino acid feeding is another critical strategy for cell growth and productivity, whilst also potentially impact the glycan profile^28^. Thus, feed strategy is important to maintain glycan synthesis and should be optimized to produce an expected glycoform. Nucleotide-sugar precursors such as uridine, glucosamine and galactose modulate intracellular nucleotide-sugar pools and resulting sialylation and antennarity levels^28^. Galactose feeding can help facilitate a more fully galactosylated N-profile^29^. We chose galactose in this experiment because its effect on glycan profile was observed in work previously done on the development of this product.

In this study, a Her2-binding antibody was developed as a biosimilar to Herceptin. Although the amino acid sequence was the same as that of the originator, the glycan profile expressed by the candidate clone in the initial culture condition was different. So, in order to explore the optimum medium combination to attain a biosimilar antibody, we designed an optimization experiment by DoE in JMP and carried out the experiment in parallel micro-bioreactor platform AMBR 15. Due to the similarity of glycan profiles between expressed antibody and reference was difficult to identify with a specific response, a cluster analysis method was introduced to enable modeling and optimization.

## Results

### Cell growth and titer

**Figure 2:**
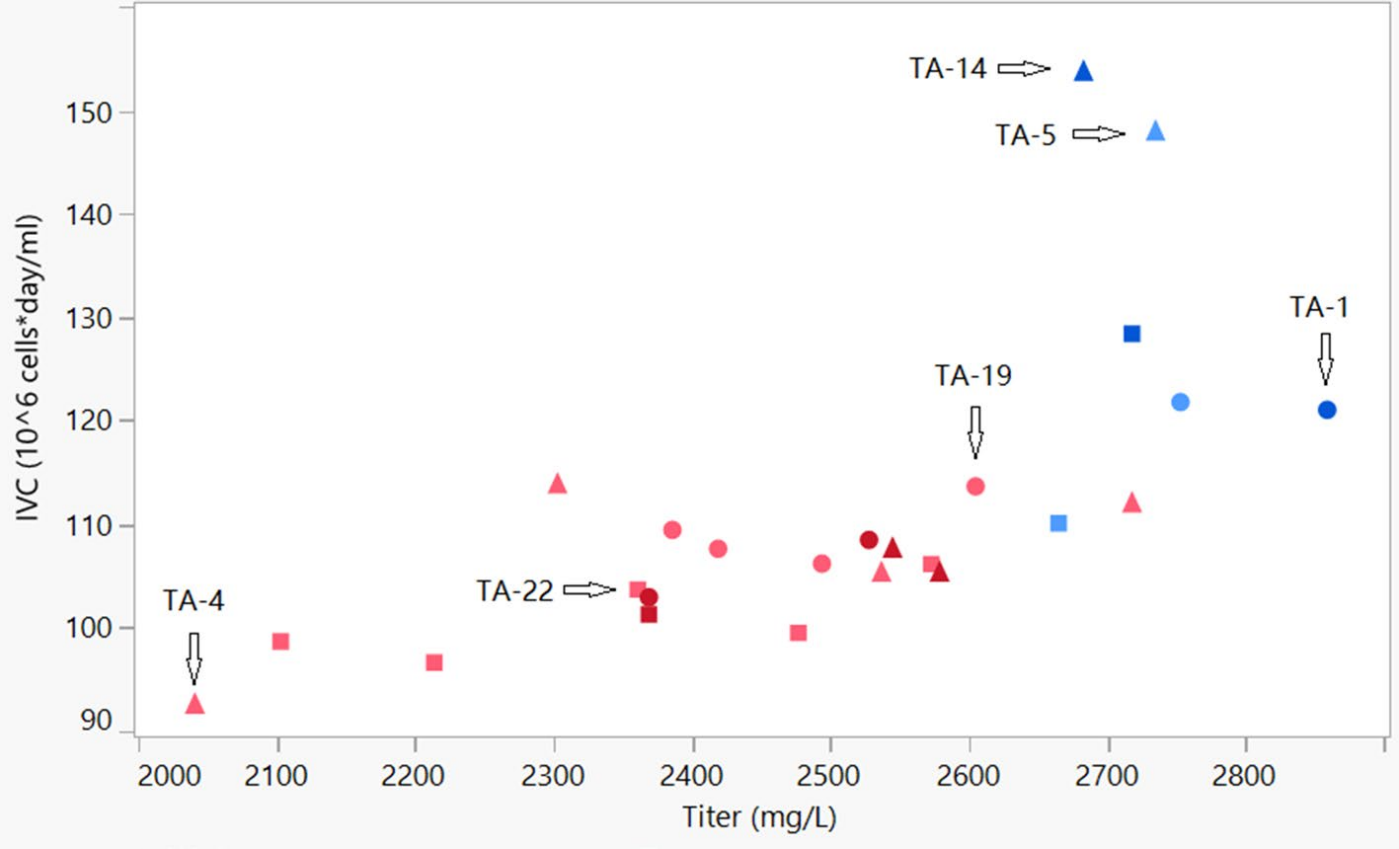
The scatter plotting of IVC vs. titer from 24-run experiment in Ambr. The IVC value indicated cell growth. Titer indicated the concentration of antibody when harvest. Basal medium CD14 is dot, basal medium CD 15 is square, Dynamis is triangle, FEED B is blue, PFF06 is red, galactose addition day 12 is light color and day 13 is dark color. The image was created by JMP V15 (http://www.jmp.com).

Medium is a vital factor to affect cell growth and productivity. 3 kinds of basal medium and 2 kinds of feed medium were tested in this study. Including feed strategy and galactose addition time, a 24-run experiment was designed by JMP software and conducted in the Ambr system. We found that the cell growth variance was significant as shown in Fig. 2. The lowest integral of the viable cell density (IVC) was 93 × 10^6^ cells·day/mL (TA-4), while the highest IVC was 154 × 10^6^ cells·day/mL (TA-14). The IVC of TA-5 and TA-14 were extraordinarily high. The basal medium and feed medium for the two runs were both Dynamis and Feed B. This composition was the best to boost cell growth among the 24 runs. But the titer of TA-5 and TA-14 were not the highest (see Fig. 2), which indicated the Dynamis and Feed B composition did not enhance the specific productivity. Considering the cost of manufacturing, titer is very important. The highest titer was 2859 μg/ml from TA-1. By observing the scatter plot (see Fig. 2), most blue spots were higher titer, which indicated that the feed medium FEED B was a good feed medium to enhance titer. To analyze the effect on titer of all factors statistically, the factors and titer data were tabulated in JMP and the regress model was fitted (Table 1). According to the ANOVA analysis, the feed medium and feeding strategy were significant to productivity, while basal medium and galactose did not impact titer significantly. FEED B and feeding strategy 1 were the optimal factor for high productivity.

**Table 1:** Statistical analysis of four factors effecting on productivity using titer value as the response.

Source	Nparm	DF	Sum of squares	F ratio	Prob > F
Base medium	2	2	57,558.08	1.9167	0.1759
Feed medium	1	1	135,926.02	9.0526	0.0075*
Strategy	1	1	185,846.72	12.3773	0.0025*
Galactose adding day	1	1	41,224.49	2.7455	0.1149

* Significant when P is less than 0.05.

### Glycosylation

**Figure 3:**
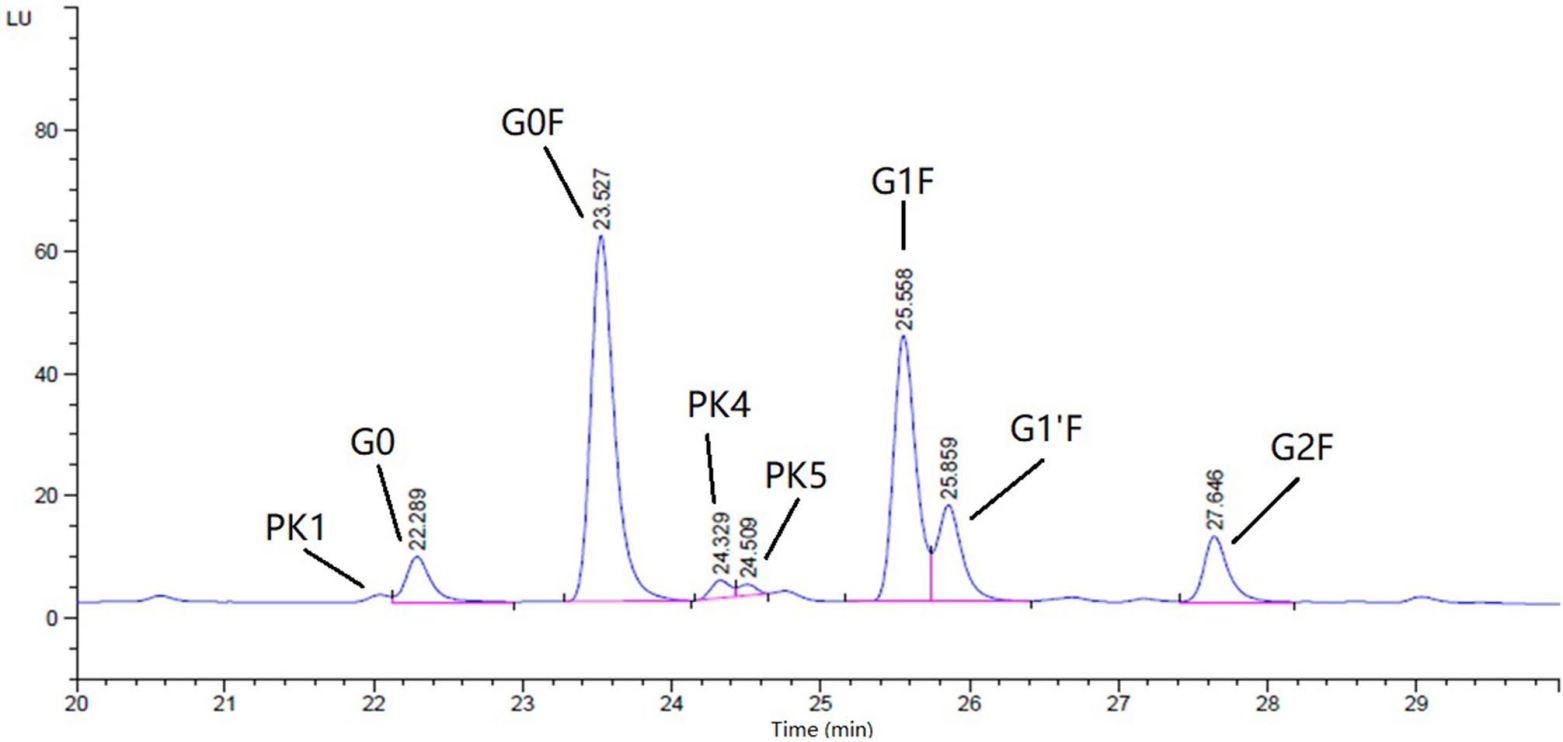
The HPLC chromatogram of glycan distribution. Compared to standards, peaks of G0, G0F, G1F, G1′F and G2F are indicated. PK1, PK4 and PK5 refers to the 3 minor unidentified peaks, respectively. The chromatogram was created by Agilent OpenLab CDS 2.0 (https://www.agilent.com/en/product/software-informatics/analytical-software-suite/chromatography-data-systems/openlab-cds).

**Figure 4:**
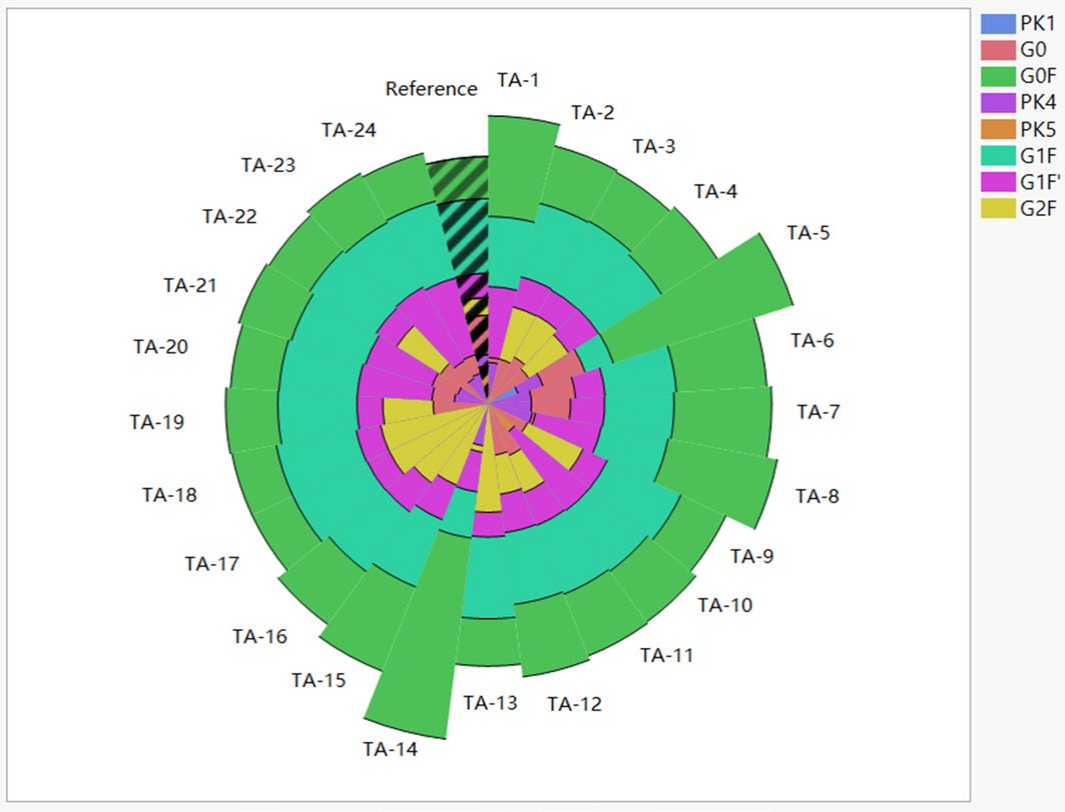
The pie chart of glycan percentage of samples and reference. TA-1 to TA-24 refers to the samples obtained from the designed 24-run experiment in Ambr. One sector represents one sample’s glycan distribution. The shadowed sector represents reference. The image was created by JMP V15 (http://www.jmp.com).

The glycosylation profiles of antibodies from the 24 micro-bioreactors were analyzed by the HPLC as described in the methodology section. The typical chromatogram of glycan distribution was shown in Fig. 3 and the G0, G0F, G1F, G1′F and G2F can be qualified comparing to the glycan standards. Three minor unidentified peaks were named as PK1, PK4 and PK5. These undefined peaks were assumed to be the same glycans of samples and reference. For the reference, the glycan of PK4 is Man5 and PK 5 is G1 as reported by Xie et al.^30^. By summarizing all of the glycan peaks of the 24 samples and reference into the pie chart (see Fig. 4), the glycosylation distribution has a wide variation, and the TA-1, TA-5 and TA-14 were significant different from other samples and reference by visual inspection. However, it is hard to identify the antibody with the most similar glycan profile to reference from the other runs.

**Figure 5:**
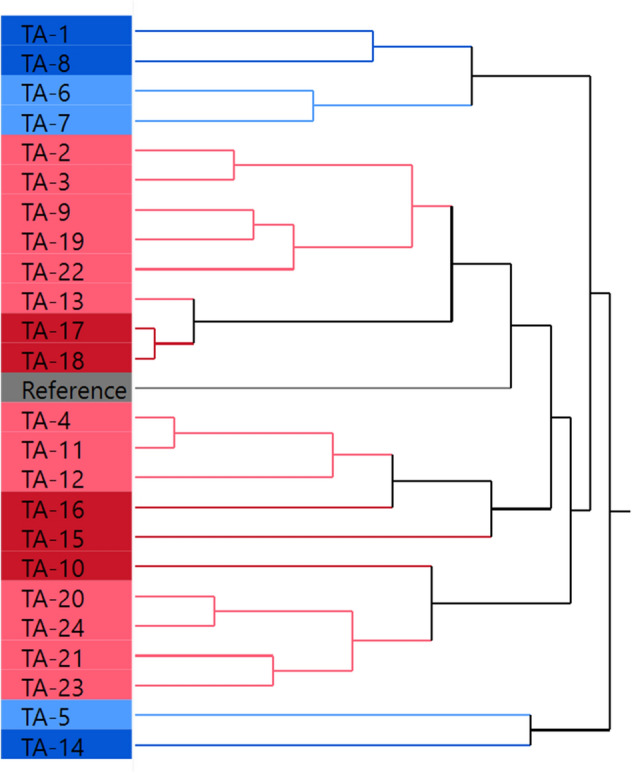
The dendrogram from hierarchical cluster analysis of samples and reference. TA-1 to TA-24 refers to the samples obtained from the designed 24-run experiment in Ambr. FEED B is blue, PFF06 is red, galactose addition day 12 is light color and day 13 is dark color. The image was created by JMP V15 (http://www.jmp.com).

**Figure 6:**
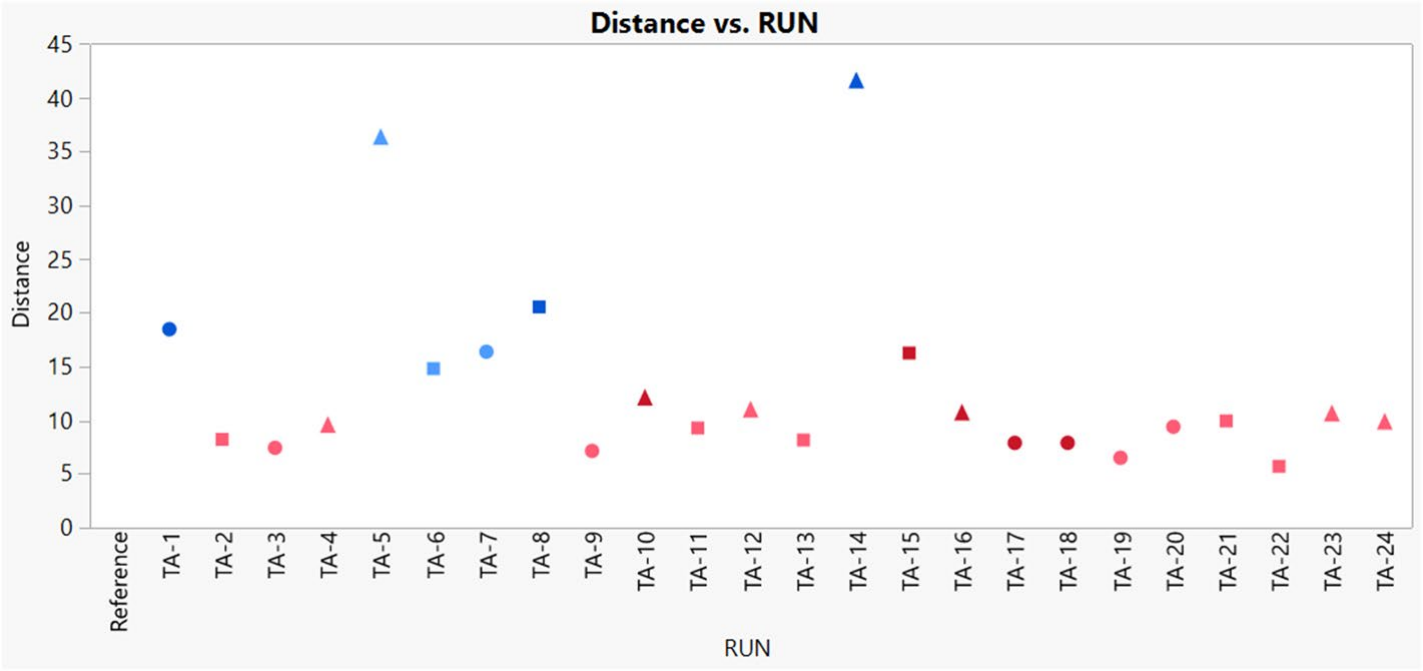
The distance between samples and reference. TA-1 to TA-24 refers to the samples obtained from the designed 24-run experiment in Ambr. Basal medium CD14 is dot, basal medium CD 15 is square, Dynamis is triangle, FEED B is blue, PFF06 is red, galactose addition on day 12 is light color and day 13 is dark color. The image was created by JMP V15 (http://www.jmp.com).

The hierarchical cluster analysis method was applied to evaluate the glycan profile similarity between the samples and the originator. A dendrogram was generated with 25 leaves including 24 samples and 1 reference (see Fig. 5). Similar glycan profiles should be in one stem. The glycosylation pattern of TA-5 and TA-14 should be different from others because they were apart on the first folk. The G0F percentage was higher than the others and the cell growth was the highest, which was caused by the medium composition of Dynamis/Feed B. The samples TA-2, 3, 9, 13, 17, 18, 19, 22 were clustered to the same group to the reference, indicating that their glycan profiles were similar to that of the reference. And samples with same color were clustered together, which indicated that feed medium and galactose addition day should have effect on glycosylation. By further checking the similarity between the samples and reference, the distance value was generated based on cluster analysis (see Fig. 6). The distance value of TA-5 and TA-14 were the highest, which was agree with observation in pie chart and cluster true. The nearest distance was 5.7 from TA-22 which should have the most similar glycan profile to reference. All of the 8 samples in same trunk with reference had the short distance less than 10.

### Factors affecting glycosylation

**Figure 7:**
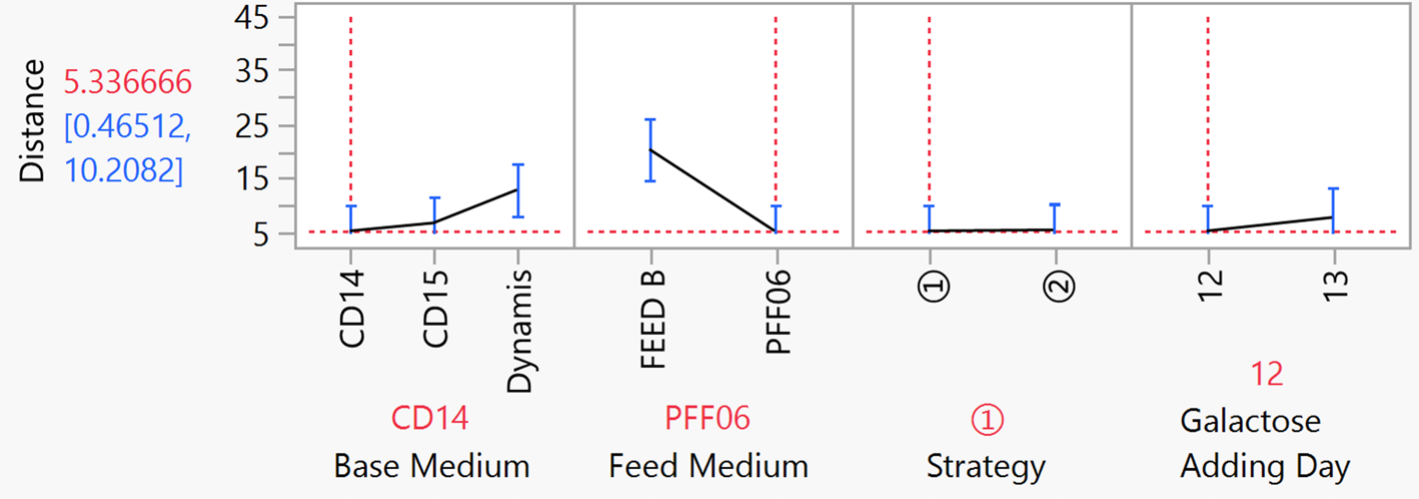
Effect evaluation of base medium, feed medium, strategy and galactose addition day on distance (Similarity). The image was created by JMP V15 (http://www.jmp.com).

To screen for factors that impact glycosylation statistically, the distance value from cluster analysis was set as the response in the model fit analysis. The statistical results were shown in Table 2. Among the four factors, base medium and feed medium had the most significant impact on similarity. On the graph of effect evaluation for factors (see Fig. 7), it is clear that the effect of CD14 and CD15 was almost the same and they were more appropriate for achieving biosimilarity for the antibody than Dynamis. For the feed medium, PFF06 can attain an antibody with more similar glycan profile to the reference compared to that with the Feed B. Consequently, the optimal medium composition was CD14 and PFF06. Considering the productivity, the feed strategy should be mode 1 and addition galactose on day 12. This condition is coincident with the run TA-19, that possessing high similarity and titer of 2605 μg/ml.

**Table 2:** Analysis of four factors effecting on glycan similarity using distance value as the response.

Source	Nparm	DF	Sum of squares	F ratio	Prob > F
Base medium	2	2	258.14567	5.1528	0.0170*
Feed medium	1	1	801.91741	32.0139	< 0.0001*
Strategy	1	1	0.18446	0.0074	0.9326
Galactose adding day	1	1	33.85210	1.3514	0.2602

* Significant when P is less than 0.05.

## Discussion and conclusions

In this study, the effect of base medium, feed medium, feeding strategy and galactose addition day on glycan profile were investigated. Medium combination was found to successfully modulate the glycosylation profile of an antibody to be similar to that of the reference. The Dynamis and Feed B combination could promote cell growth, but the glycosylation pattern was found to be different from the others. Thus, the metabolite pattern may be different from the other condition. Even though the base medium was the same, the feed medium played an important role in affecting the ratio of G0F to G1F. Andersen et al. found that galactose feeding can help to facilitate a more fully galactosylated N-glycan profile^29^. However, the galactose addition on day 12 and 13 made no significant difference to the final percentage of galactosylation. The reason may be the slow synthesis rate in the later phase and the galactosylation level reached a “plateau” at approximately 50–60%^31^.

The cluster analysis was found to be very useful in assessing the similarity between the biosimilars and the originator. Samples with similar quality could be grouped together to visualize the similarity. The specific value-distance denoting the similarity made the model fit possible, thereby the factors’ impact on similarity can be evaluated by ANOVA statistically.

Glycosylation is a vital CQA for monoclonal antibodies and recombinant proteins because of its impact on efficacy and pharmacokinetics. Modulation of glycan profile is often used in developing biosimilar products. Statistical method such as the DoE is very useful method to optimize or adjust process parameters for the purpose of increasing or decreasing a specific response. However, glycosylation profile is not a single value like a titer measurement, so it is hard to apply DoE method directly. In order to solve this problem, cluster analysis can be introduced. By using this method, the similarity of glycan between the samples and reference was demonstrated as the specific value-distance. In this study, base medium and feed medium were found to be the significant factors in impacting the glycosylation. A combination of medium and feed strategy was developed to attain the most similar glycoprotein to the originator. The best condition was found to be using CD14 as the base medium, PFF06 as the feed medium, feeding as strategy 1 and addition galactose on day 12.

Besides the glycosylation profile, other quality attributes such as cIEF, CEX and peptides mapping are of multi-parameter format. Thus, the cluster analysis method introduced here could also help to assess similarity of these quality attributes. Likewise, DoE can be used to evaluate factor effect and optimize inputs leading to reduced time and cost.

## Material and methods

### Cell line and reagents

A DG44 derived CHO cell producing Her2-binding antibody was studied in this article. The basal medium was CD14 (CD14, OPM Biosciences), CD15(CD15, OPM Biosciences) and Dynamis (A26615, Thermofisher) supplemented with 6 mM Glutamine (G8540, Sigma). Feed medium were EfficientFeed B (FEED B, A12456, Thermofisher) and PFF06 (PFF06, OPM Biosciences). The additive was galactose (G5388, Sigma). The compositions of all the used medium were developed by their vendors.

### Cell culture condition and experimental design

The candidate cell line was cultured in Dynamis in clone screening phase. To test the new medium, the cells were cultured in CD14 and CD15 for 3 passages to allow the cell to adapt to the new medium. The fed-batch culture mode was applied in AMBR with temperature, pH, DO and agitation controlled. The temperature was set at 37 °C on the beginning culture and shifted to 35 °C on day 4. DO was maintained at 50% and pH was in the range of 6.8–7.2. The seeding density was 1 million cells/mL with 12 mL initial culture volume. Two feeding strategies was tested in the experiment. For strategy 1, feeding started from day 3 and the feeding volume was calculated through Eq. (1). For strategy 2, feeding volume was 4% of the initial volume on day 3 and 6% of the initial volume every other day till the harvest day. The glucose residual concentration was tested every day and glucose was supplemented to 3 g/L when viable cell density (VCD) was less than 10 million cells/mL and to 4 g/L otherwise. The feeding volume of glucose was calculated through Eq. (2). The base medium, feed medium, feeding strategy and the galactose addition day were designed by custom DoE function as four factors using JMP. 24 runs of experiment were conducted according to the design (Table 3). All the runs were harvested on day 14.

1 $${\text{V}}_{{{\text{feed}}}} = {\text{V}}_{{{\text{current}}}} *{\text{Q}}_{{{\text{feed}}}} *\left( {{\text{VCD}}_{1} + {\text{VCD}}_{2} } \right)/2/{\text{Glc}}_{{{\text{feed}}}} /1000$$

2 $${\text{V}}_{{{\text{Glc}}}} = \left( {\left( {{\mkern 1mu} {\text{Glc}}_{{{\text{target}}}} - {\text{Glc}}_{{{\text{test}}}} } \right)*{\text{V}}_{{{\text{current}}}} + \left( {{\mkern 1mu} {\text{Glc}}_{{{\text{target}}}} - {\text{Glc}}_{{{\text{feed}}}} } \right)*{\text{V}}_{{{\text{feed}}}} } \right)/{\mkern 1mu} {\text{Glc}}_{{{\text{solution}}}}$$

$${\text{Q}}_{{{\text{feed}}}} = 75\;{\text{g}}/10^{9} \;{\text{cells}}$$

$${\text{If VC}}{{\text{D}}_1} < 1.0 \times {10^7}{\text{cell/mL}}, \, {\text{Glc}}{_{{\text{target}}}} = 3{\text{ g/L, or else }}{\text{Glc}}{_{{\text{target}}}} = 4{\text{ g/L}}$$

$${\text{If VC}}{{\text{D}}_1} < 1.0 \times {10^7}{\text{cell/mL}},{\text{ VC}}{{\text{D}}_2} = e\left( {0.02*24} \right)*{\text{VC}}{{\text{D}}_1},{\text{ or else }}{\text{VCD}}{_2} = {\text{VCD}}{_1}$$

**Table 3:** The design of experiment for 24-run experiment in Ambr.

RUN	Base medium	Feed medium	Strategy	Galactose adding day
TA-1	CD14	FEED B	①	D13
TA-2	CD15	PFF06	②	D12
TA-3	CD14	PFF06	①	D12
TA-4	Dynamis	PFF06	②	D12
TA-5	Dynamis	FEED B	①	D12
TA-6	CD15	FEED B	①	D12
TA-7	CD14	FEED B	①	D12
TA-8	CD15	FEED B	①	D13
TA-9	CD14	PFF06	②	D12
TA-10	Dynamis	PFF06	①	D13
TA-11	CD15	PFF06	①	D12
TA-12	Dynamis	PFF06	①	D12
TA-13	CD15	PFF06	①	D13
TA-14	Dynamis	FEED B	①	D13
TA-15	CD15	PFF06	②	D13
TA-16	Dynamis	PFF06	②	D13
TA-17	CD14	PFF06	②	D13
TA-18	CD14	PFF06	①	D13
TA-19	CD14	PFF06	①	D12
TA-20	CD14	PFF06	②	D12
TA-21	CD15	PFF06	①	D12
TA-22	CD15	PFF06	②	D12
TA-23	Dynamis	PFF06	①	D12
TA-24	Dynamis	PFF06	②	D12

V_feed_: Feed volume of current day (mL), V_current_: Culture volume before feeding (mL), V_Glc_: Glucose solution addition volume (mL), Q _feed_: Feed rate factor, VCD_1_: Viable cell density of current day (10^6^ cell/mL), VCD_2_: Calculated viable cell density of next day (10^6^ cell/mL), Glc_feed_: Glucose concentration in feed medium (g/L), Glc_test_: Glucose concentration in culture(g/L), Glc_target_: Target glucose concentration after feeding (g/L), Glc_solution_: Concentration of glucose solution (g/L).

### Monitoring of cell growth and titer

VCD and viability were measured with CounterStar IC1000 every day. To compare cell growth for the 14-day culture in fed-batch, the IVC was calculated through Eq. (3). The antibody titer was tested by CEDEX Bio (Roche) after centrifugation of the sample for 5 min at 3000 rpm.

3 $${\text{IVC}}={\sum }_{k=1}^{n}({\text{VCD}}\left({\text{k}}\right)+{\text{VCD}}\left({\text{k}}-1\right))/2$$

VCD(K) Viable cell density of day K, *n* Harvest day.

### Purification and analysis of the glycosylation profile

The antibody was captured from the cell harvest supernatant using protein A column (Mabselect SuRe, GE) at first. After capture completed, the target protein was eluted by 20 mM citrate buffer (pH 3.60, Sinopharm). Elution was collected from 100 mAU and ended when the absorbance value come back to 100 mAU. The reference product was Trastuzumab purchased from Asian market. The N-glycans were released from 300 μg antibody by digestion with 0.5 μL PNGaseF (PO704L, Biolabs NEB) followed by labeling with 6 μL 2-AB (76,884, Sigma) at 65 °C for 3 h. Glycosylation patterns were analyzed by HPLC (1260, Agilent) with AdvanceBio glycan mapping column (4.6 mm × 150 mm, Agilent) and fluorescence detector (Ex: 260 nm, Em: 430 nm). The solvent A was 100 mM ammonium formate (17843, Sigma Fluka) and solvent B was acetonitrile (A998-4, Fisher). The gradient conditions of A:B used were 25:75 on 0 min, 40:60 on 25 min, 100:0 on 30 min, 25:75 on 35 min to 40 min. Elution rate was 0.5 mL/min and sample volume was 2 μL. The distribution of different major glycans such as G0F, G1F, G1′F and G2F can be shown in the chromatography map. Area under the curve analysis was done by integrating the peaks from 16 to 30 min followed by calculating the area percentage for each peak by area normalization method using the HPLC software.

### Similarity assessment and ANOVA

The peak area percentages from the glycan analysis are used for assigning the similarity in this case. Peaks area percentages of samples and reference were entered to form a symmetrical matrix and Hierarchical Cluster Analysis was performed using JMP software (SAS Institute Inc.). A dendrogram can be generated and the distance matrix was saved to another data table. The distance denotes the similarity to originator. To analyze the effect of the 4 factors on glycan similarity, the distance was set as the response value and then fit model was run in JMP.

## Data Availability

All data generated or analyzed during this study are included in this published article.
